# Supplementary material for: Simple Synthesis of Cobalt Carbonate Hydroxide Hydrate and Reduced Graphene Oxide Hybrid Structure for High-Performance Room Temperature NH3 Sensor
Source: Sensors (Basel). 2019 Feb 1;19(3):615. doi: 10.3390/s19030615 (PMC6387293; doi:10.3390/s19030615)
Supplement: Supplementary file 1 [file sensors-19-00615-s001.pdf]

Supporting Information

1. The control and calibration of ammonia concentration.

In this work, we used the ammonia solution instead of ammonia gas. We obtained the concentration of ammonia by the volume of ammonia solution extracted and the volume of the chamber. The specific calculation was as follows. The concentration of ammonia ($C_{ammonia}(ppm)$) was calculated by Equation 1,

$$C_{ammonia} = V_{ammonia}/V \quad \text{S-1}$$

Where, V was the total volume of chamber (4.67 L), $V_{ammonia}$ was the volume of ammonia gas at normal temperature and pressure, which can be obtained according to clausius-clapeyron equation as follows,

$$PV_{ammonia} = nRT \quad \text{S-2}$$

R was a gas constant. P, T represents air pressure and temperature, respectively. n was the amount of substance, which was obtained by the following equation,

$$n = v \cdot \frac{wt.\% \cdot \rho}{M} \quad \text{S-3}$$

Where, v was the volume of ammonia solution tested, M was the relative molecular mass of ammonia, $wt.\%$ and ρ represented the mass fraction and density of ammonia solution.

2. Gas sensing detection of CCHH and CCHH-RGO-0.1

Due to the resistance of these two sensors being too large to exceed the test precision of the multimeter (2000, Keithley Instruments), the test was conducted with semiconductor parameter analyzer 4155C.

As can be seen from the Figure 1s, the signal current of CCHH can only reach 40nA at a fixed test voltage of 20V. Such a small signal current will cause the test results to be disturbed by environmental noise as shown in curve fluctuation in the picture.

Similar to CCHH, extremely high resistance of CCHH-RGO-0.1 leads to ultra-low signal current, which leads to the introduction of environmental noise shown in the Figure 2s. The difference is that environmental noise is partially suppressed by introducing a small amount of RGO. As can be seen from the Figure 1s and Figure 2s, the test curve fluctuation of CCHH-RGO-0.1 is much smaller than that of CCHH.

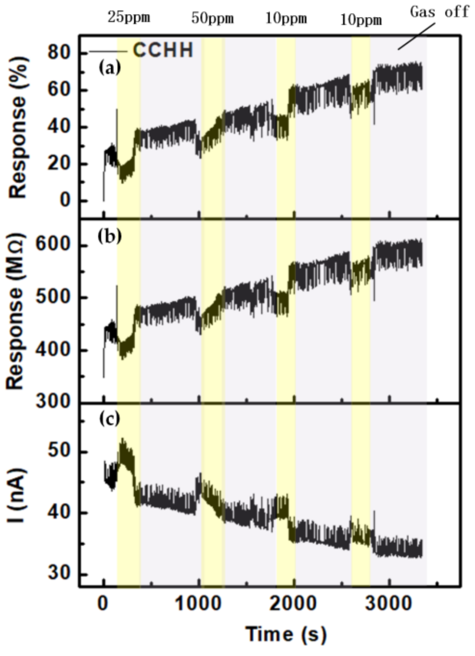

Figure S1. Gas sensing (**a**) response, (**b**) resistance response, (**c**) signal current response of CCHH to different concentrations of ammonia.

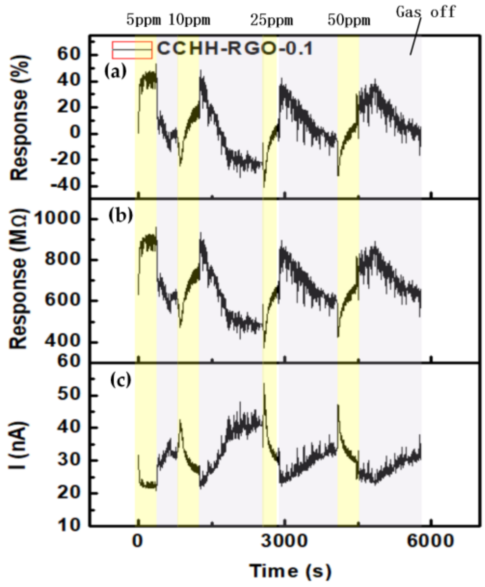

Figure S2. Gas sensing (**a**) response, (**b**) resistance response, (**c**) signal current response of CCHH-RGO-0.1 to different concentrations of ammonia.
